# Work and Pay

**Published:** 1909-11-27

**Authors:** B. Burford Rawlings


*To the Editor of* The Hospital.

### WORK AND PAY.

*To the Editor of* The Hospital.

Sir,—I have no desire to reopen in your columns the brief correspondence, not begun by me, printed in the *Hospital Gazette*, upon which you comment in your current number. I would rather ask permission to deal with the conclusions you draw. I submit that they are wanting in due perception of the distance which separates the standpoints of the two men whom you describe as "contestants." You write as though they had occupied precisely similar positions, had breathed the same mental atmosphere, and had been influenced by identical processes of thought; whereas, without invoking the aid of their own utterances, it would be easy, by setting forth actual figures, to demonstrate mathematically the fallacy of this assumption.

But such methods would be disagreeable. I prefer to dwell for an instant upon the essential differences in the office held by the one and the other. Put shortly, I have no successor. From the beginning of my service the office I held was charged by the by-laws with a general responsibility of control from which the secretaryship is now formally exempted, while during the last sixteen years, these responsibilities, somewhat enlarged, were recognised in the title of "Director." On the day I left the hospital my office ceased to be, and the authority of the secretaries subsequently appointed was limited strictly to their own department.

Apart from this, we must remember that I had been for some twelve years or more a fellow-worker with the actual founders, that I imbibed something of their enthusiasm, was a witness of the uninterrupted growth of the institution in each of its several divisions, and an increase of accommodation for in-patients from thirty to two hundred beds, a total not since added to. The records of the hospital show (1) that the increase of remuneration of the secretarial office did not correspond with this growth, (2) that the title of "Director" carried no additional salary, and (3) that all the work in connection with the rebuilding of the hospital and its Finchley branch, including the collection of funds, was performed gratuitously, and is so acknowledged in the reports.

In these circumstances, is it unreasonable that I should break my long silence under the attack made upon me by one who would be regarded as well-informed, seeing that he has access to all the hospital documents, and, among them, to some of which an even cursory examination would establish the exact opposite of his insinuations?

But if, Sir, I refuse to accept this gentleman's judgment or to admit his right or his competence to judge me, I protest still more, because you write with authority, against the doctrine set forth in your article that "no good man is justified in continuing to work for a great hospital if he is not adequately paid for his services." As we see, differences of view obtain among those most concerned. Each must judge for himself. And, personally, I resent, as I have resented always, the suggestion that a man who receives a salary is *ipso facto* placed outside the pale, and has no part in the philanthropy of his institution. There is no such necessity, and to assert the contrary seems to me to inflict a grievous wrong upon many a worker.

What satisfaction I now derive from the memory of my work in Queen Square comes chiefly from the fact that I was privileged to devise and to carry through many laborious undertakings without a thought of personal advantage, and to me it appears the degradation of an honourable office which should be attractive to men not perhaps able to dispense altogether with salary, but who are yet capable of unselfish ambitions, to adopt the view that the occupier is not "justified" in doing more than he is paid for.

For my own part, I could desire no better vindication of my attitude than the article from The Hospital which was reproduced in the *Times* of July 20, 1901. That it should be necessary at this late period for me to begin to write about myself I regard as a misfortune. Throughout the whole course of the hospital literature produced during my term of office I do not think one allusion to me will be found. The peccant paragraph in the article I innocently contributed to the *Hospital Gazette* referred, not to me, but to others, some of whom I have known personally, some only by reputation, and while we must of course believe that there is one secretary at least who despises the altruistic possibilities of his position, I believe that it is no more than a commonplace of truism to assert that were it not for the devotion of some so-called "paid officials," the public philanthropy of the country would lose a great part of its distinction, and the voluntary method of maintaining hospitals would have been superseded long ago. I am, Sir, your obedient servant,

November 22, 1909. B. Burford Rawlings.

